# Central Venous Pressure as a Predictor of Acute Kidney Injury in Cardiac Surgery: A Systematic Review of Observational Studies

**DOI:** 10.3390/diagnostics15050530

**Published:** 2025-02-21

**Authors:** Panagiota Griva, Vasiliki Griva, Dimitra Samara, Christina Talliou, Konstantina Panagouli, Loizos Roungeris

**Affiliations:** 1 Department of Anesthesiology, University General Hospital Attikon, 12462 Athens, Greece; kpana@med.uoa.gr; 2 Department of Internal Medicine, General Hospital of Athens “Sismanoglio”, 15126 Athens, Greece; vasiagriva@med.uoa.gr; 3 School of Medicine, National and Kapodistrian University of Athens, 11527 Athens, Greece; dimitrasam@med.uoa.gr (D.S.); christall@med.uoa.gr (C.T.); 4 Department of Anaesthesiology, Rea Maternity Hospital, 17564 Athens, Greece; lrounger@med.uoa.gr

**Keywords:** central venous pressure, acute kidney injury, cardiac surgery, fluid resuscitation, hemodynamic monitoring

## Abstract

**Background/Objectives**: Acute kidney injury (AKI) is a syndrome characterized by impaired kidney function, which is associated with reduced survival and increased morbidity. Central venous pressure (CVP) is a widely used hemodynamic parameter for assessing the volume status of patients and evaluating their response to fluid resuscitation. This systematic review aims to analyze various prospective and retrospective observational and controlled trials to determine the association between CVP and the risk of developing AKI in patients undergoing cardiac surgery. Additionally, it examines whether elevated CVP serves as an accurate predictor of AKI in this patient population. **Methods**: A systematic review was conducted following the PRISMA (Preferred Reporting Items for Systematic Reviews and Meta-Analyses) guidelines, using PubMed as the primary database. The search focused on studies published after 2014 that included adult patients undergoing cardiac surgery with reported measurements of CVP and kidney function assessment. Studies conducted on animals, pediatric populations, those published before 2014, or in languages other than English were excluded from the review. **Results**: Through the analysis of 21 studies, a clear association between higher CVP and increased AKI risk emerged. The most critical CVP thresholds identified were 10 mmHg, 12 mmHg, 14 mmHg, and 20 mmHg, with risk increasing progressively beyond these values. CVP ≥ 10 mmHg was the most commonly reported cutoff for elevated AKI risk, showing 1.42 to 4.53 times increased odds. CVP ≥ 12 mmHg further amplified the risk, while CVP ≥ 14 mmHg was consistently associated with severe AKI and the need for RRT. The highest threshold (CVP ≥ 20 mmHg) showed the greatest risk escalation, linked to fluid overload, right heart failure, and mortality. Studies also suggest an optimal CVP range of 6–8 mmHg to minimize AKI incidence. **Conclusions**: Elevated CVP is an independent risk factor for the development of AKI in patients undergoing cardiac surgery. These findings suggest that CVP monitoring can play a significant role in predicting AKI and guiding perioperative management strategies.

## 1. Introduction

Acute kidney injury (AKI) is a syndrome of impaired kidney function associated with reduced survival and increased morbidity. AKI is determined by one of the below characteristics: (1) serum creatinine increase by ≥0.3 mg/dL within 48 h, (2) serum creatinine elevated over 1.5 times from baseline during the last 7 days, or (3) urine output < 0.5 mL/kg/h for 6 h [1]. The severity of AKI can be determined using various classification systems, such as the KDIGO (Kidney Disease Improving Global Outcomes) staging system (Table 1), the RIFLE system (Table 2), and the AKIN (Table 3) classification system.

AKI is a significant complication in acute conditions, such as stroke, where patients with preexisting chronic kidney disease (CKD) face heightened risks of adverse outcomes. As described by Rajesh et al. [2], CKD contributes to cerebrovascular pathology through systemic inflammation, oxidative stress, endothelial dysfunction, vascular calcification, and impaired cerebral autoregulation, all of which exacerbate stroke severity and influence treatment responses. The interplay between CKD and AKI, often described as the “acute-on-chronic” phenomenon, underscores the vulnerability of these patients to further renal impairment due to hemodynamic instability, inflammatory responses, and neurohormonal dysregulation. In critically ill patients, including those with acute stroke, AKI can worsen systemic dysfunction, prolong hospitalization, and increase mortality. Furthermore, CKD-related alterations in fluid dynamics and endothelial function may modify the relationship between central venous pressure (CVP) and AKI risk, highlighting the need for tailored management strategies. Given the bidirectional relationship between stroke and CKD, optimizing care requires integrating renal considerations into treatment protocols, particularly when utilizing reperfusion therapies such as thrombolysis or endovascular thrombectomy, which may further impact renal function.

The renal system exhibits a heightened vulnerability to ischemic damage amidst cardiac surgical procedures [3]. During cardiopulmonary bypass (CPB), there exists a compromised renal oxygen supply/demand ratio attributable to the renal vasoconstriction and hemodilution occurring during and in the immediate aftermath of CPB [4]. Cardiac surgery-related AKI is estimated to manifest in approximately 20 to 30% of patients undergoing such procedures [5]. Comorbid conditions, such as CKD, diabetes mellitus, and heart failure, significantly contribute to the elevation of AKI risk, particularly within the perioperative period. CKD predisposes individuals to AKI due to diminished renal reserve, endothelial dysfunction, and an increased vulnerability to hemodynamic variations. Similarly, diabetes mellitus augments the likelihood of AKI through the mechanisms of chronic inflammation, microvascular injury, and oxidative stress, which can intensify renal damage post-surgery. Heart failure exacerbates this risk by modifying renal perfusion and elevating CVP, resulting in venous congestion and impaired renal function. In light of these considerations, the incorporation of comorbidities into perioperative risk assessment is imperative for the identification of patients at high risk and the enhancement of preventive measures aimed at reducing the occurrence and severity of AKI. Several scoring systems are used in clinical practice to predict AKI after cardiac surgery. For example, the Cleveland Clinic Score (Table 4) and the Mehta Score (Table 5) have been developed to assess AKI risk in cardiac surgery patients.

The hemodynamic regulation of Renal Blood Flow (RBF) and renal venous pressure are key determinants of renal function. Blood pressure is used as an index for organ perfusion [6]. Certainly, the maintenance of normal blood pressure is of major importance to avoid AKI. Although Mean Arterial Pressure (MAP) is used commonly for this purpose, it is not an accurate indicator [7]. Sato et al. [8] proved that there is no association between MAP and AKI, but the elevation of CVP is related to a higher incidence of AKI. In the context of cardiac surgeries, CVP is particularly valuable as an essential hemodynamic parameter for assessing a patient’s volume status and responsiveness to fluid administration during both the intraoperative and postoperative phases. Moreover, CVP plays a critical role in determining the filling pressure and preload of the right ventricle, which directly impacts stroke volume—a key determinant of cardiac output (CO) [9]. Guinot et al. [10] observed that impaired renal function in cardiac surgery patients was associated with early postoperative vena cava dilatation and elevated CVP. To achieve the most favorable patient outcomes, especially in the context of postoperative recovery following cardiac surgery, it is imperative to prevent prolonged elevations in CVP, as such conditions correlate with a heightened likelihood of adverse events, including renal impairment.

The present review aimed to study various prospective and retrospective observational and control trials to analyze the association between CVP and the possibility of AKI. More specifically, this study aimed to prove whether elevated CVP is an accurate indicator for the prediction of AKI in patients undergoing cardiac surgery. Central venous pressure is a crucial hemodynamic parameter in the context of cardiac surgery due to its direct relationship with right heart function, venous return, and fluid balance. Unlike MAP or the cardiac index (CI), which primarily reflect systemic perfusion and cardiac output, CVP provides specific insights into venous congestion and right ventricular performance—both of which play a significant role in AKI development. During cardiac surgery, factors such as cardiopulmonary bypass, fluid shifts, and perioperative inflammation can lead to elevated CVP, increasing renal venous pressure and impairing kidney perfusion. This venous congestion mechanism is particularly relevant to AKI pathophysiology in surgical settings. While other parameters contribute to overall hemodynamic assessment, CVP uniquely captures the interplay between volume status and right heart function, making it a valuable predictor of AKI risk in this patient population [10]. A comprehensive and systematic synthesis of the prevailing evidence is imperative to elucidate whether CVP functions as a dependable prognostic indicator of AKI within this distinct cohort, establish optimal CVP thresholds, and examine the interrelations between CVP, cardiopulmonary bypass-induced inflammation, and renal outcomes.

## 2. Materials and Methods

A systematic literature review using Preferred Reporting Items for Systematic Reviews and Meta-Analyses (PRISMA) guidelines was performed from November 2024 to January 2025. The research included articles published in PubMed after 2014 and was focused on the following terms: “central venous pressure”, “acute kidney injury”, and “cardiac surgery”. This systematic review adhered to a predetermined protocol that was previously published in the PROSPERO database (CRD42024620199). The review protocol is registered in the PROSPERO database.

### 2.1. Inclusion Criteria

The inclusion criteria were defined considering “Patients, Intervention, Comparator, Outcomes, and Study Design” (PICOS), as follows: (1) population: adult patients over 18 years old undergoing cardiac surgery; (2) intervention: measurement of CVP and assessment of kidney function; (3) outcomes: the occurrence of AKI; (4) study design: prospective and retrospective cohort studies after 2014.

### 2.2. Exclusion Criteria

Experimental studies in children, experimental studies in animals, studies examining non-cardiac surgery, studies before 2014, reviews, systematic reviews with meta-analyses, post hoc analyses, and studies in a different language other than English were excluded.

### 2.3. Research Strategy

**Figure 1:**
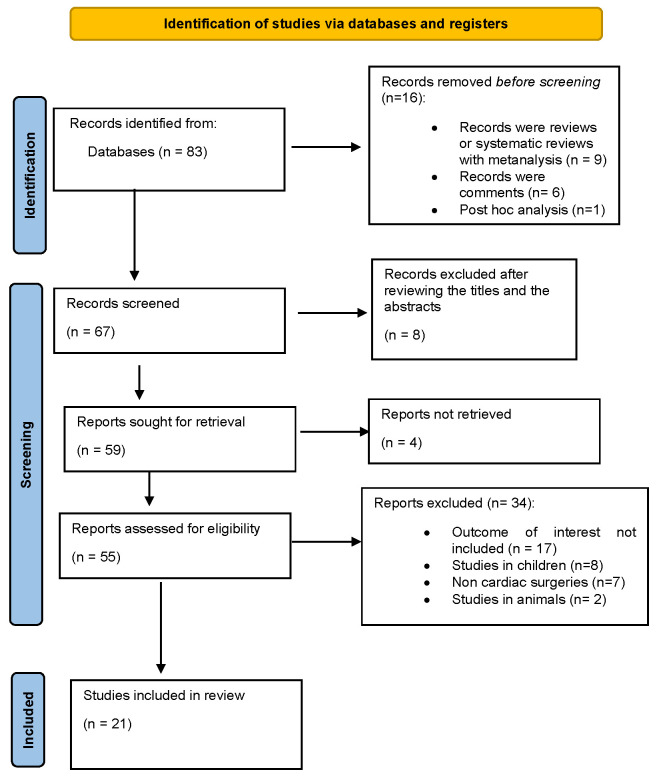
Flow diagram of PRISMA.

Articles were retrieved using the literature management software Mendeley (Version 1.19.5, Elsevier, Amsterdam, Netherlands), which facilitated searches in the PubMed and Cochrane Library databases. The initial search yielded 83 publications related to the development of AKI during cardiac surgeries and elevated CVP. After removing 16 records that were reviews and systematic reviews with metanalyses (*n* = 9), comments (*n* = 6), and post hoc analyses (*n* = 1), 67 studies were initially screened and reviewed via the title and abstract. The studies were initially screened and reviewed via the title and abstract by the second (V.G.), fifth (K.P.), and sixth (L.R.) authors. Eight studies were excluded after reviewing the titles and abstracts and four reports were not retrieved. Fifty-five studies were suitable for the initial review. After excluding animal trials (*n* = 2), studies in children (*n* = 8), and non-cardiac surgeries (*n* = 7), 38 studies were deemed suitable for the preliminary review. Full-text articles were then further screened, resulting in the exclusion of an additional 17 studies because their endpoints did not align with the search criteria. Full-text articles were then screened by the first (P.G.), third (D.S.), and fourth authors (C.T.). Ultimately, 21 studies were included in the review. Figure 1 provides a flow diagram illustrating the screening process.

### 2.4. Data Extraction and Analysis

The studies included in the review had varying designs (prospective, retrospective) and encompassed a range of cardiac surgeries, including coronary artery bypass grafting, heart valve surgery, surgeries on the ascending aorta, and transcatheter aortic valve implantation. The patient populations were heterogeneous, including individuals with comorbidities such as chronic kidney disease, diabetes, and heart failure. To ensure consistency in the application of AKI definitions across studies, we conducted a thorough review of the methodologies used in the included studies. Only those studies that utilized well-established AKI classification systems, such as KDIGO, RIFLE, and AKIN, were included in the review. For each study, the specific AKI definition applied was documented and reviewed in detail. The table below (Table 6) provides a summary of the AKI definitions used in each study, including the relevant criteria for diagnosis and staging.

### 2.5. Bias Assessment

**Figure 2:**
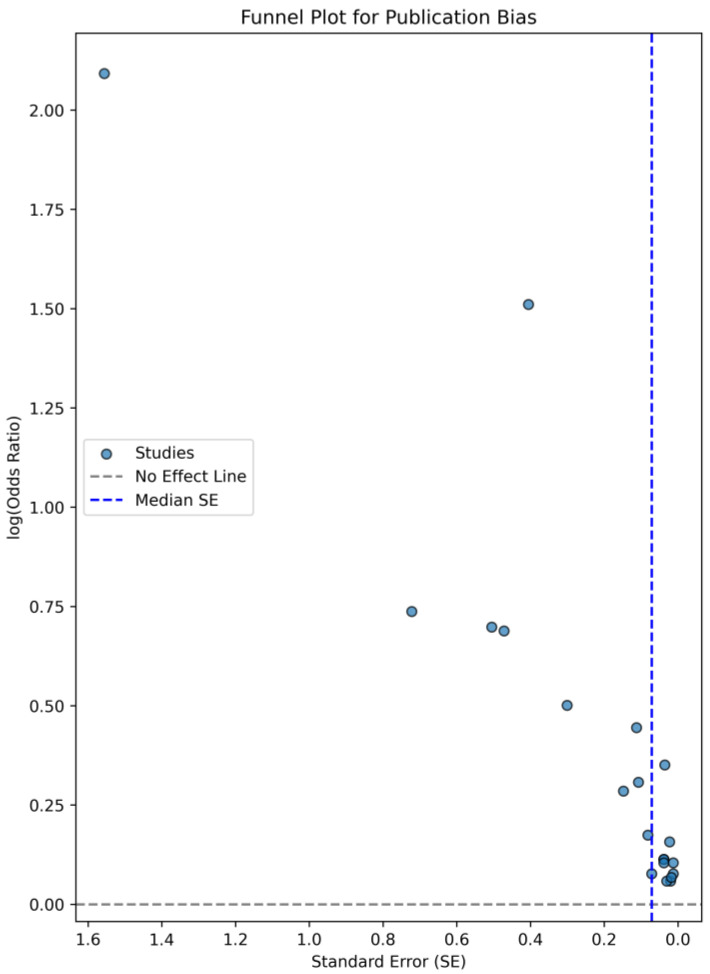
Funnel plot.

For the purpose of evaluating the quality of the studies incorporated within the systematic review, the Newcastle–Ottawa scale (NOS) was employed. This scale is explicitly designed for the assessment of non-randomized observational studies. The evaluation of studies is conducted through a star rating system, which permits a maximum allocation of nine stars, contingent upon the selection of observational cohorts, the comparability of the study groups, and the pertinent outcomes. Based on the findings, one study attained a score of 5 stars, four studies achieved 6 stars, nine studies garnered 7 stars, five studies attained 8 stars, and two studies achieved 9 stars. To assess publication bias, we generated a funnel plot (Figure 2) and conducted Egger’s test. Visual inspection of the funnel plot suggested asymmetry, and Egger’s test yielded a *p*-value of 1.90 × 10^−8^, indicating potential publication bias. This suggests that smaller studies with negative or non-significant findings may be underrepresented in the literature.

## 3. Results

The reports focus on the measurement of CVP in patients who have undergone cardiac surgery in order to assess the probability of AKI. According to several reports, the elevation of CVP is an accurate, independent predictor for the development of AKI (Table 6).

### 3.1. Predicting AKI in Relation to CVP

Yang et al. [11] identified that, after cardiac surgery, patients with CVP values above 10 mmHg had a 6-fold higher occurrence of AKI than patients with CVP below 10 mmHg. In their study, patients were divided into high-CVP (>10 mmHg) and low-CVP (<10 mmHg) groups. Among those in the high-CVP group, 347 patients (43.32%) developed AKI, compared to 86 patients (7.54%) in the low-CVP group, with the difference being statistically significant (*p* < 0.0001). This observation is further corroborated by the study conducted by Li et al. [12], which included 5533 eligible participants who underwent cardiac surgery. Their research elucidated that the probability of AKI augments in direct correlation with elevated postoperative CVP levels. In particular, subjects exhibiting a postoperative CVP of ≥10 mmHg presented with an adjusted odds ratio of 4.53 for the occurrence of AKI and 8.42 for the incidence of severe AKI. Beaubien-Souligny et al. [13] prospectively recruited 145 patients for their study, of whom 49 (33.8%) developed AKI following cardiac surgery. Higher CVP measurements at the conclusion of surgery were significantly associated with the development of AKI, with a hazard ratio (HR) of 1.04 (95% CI: 1.01–1.08; *p* = 0.02) per 1 mmHg increase in CVP.

Chen et al. [14] analyzed 5127 participants, of whom 1,070 (20.9%) developed AKI and 327 (7.2%) progressed to acute kidney disease (AKD). CVP thresholds (≥12, ≥16, and ≥20 mmHg) were pre-defined based on intraoperative venous congestion variability. Sustained CVP elevations were significantly linked to AKD, with hazard ratios per 10 min duration: CVP ≥ 12 mmHg (HR = 1.03), CVP ≥ 16 mmHg (HR = 1.04), and CVP ≥ 20 mmHg (HR = 1.07). Each 10 min duration above these thresholds increased AKI odds by 3%, 6%, and 13%, respectively, highlighting the risks of prolonged venous congestion. Lopez et al. [15] quantified venous congestion as the area under the curve (AUC) for CVP values exceeding 12 mmHg, based on the typical CVP range of 0–10 mmHg in spontaneously breathing humans and the elevated CVP caused by positive pressure ventilation during cardiac surgery. Among 425 patients, the median CVP at catheter placement was 13 mmHg, and 104 developed AKI (per KDIGO criteria). The venous congestion AUCs were 273 mmHg·min for CVP > 12 mmHg, 66 mmHg·min for CVP > 16 mmHg, and 11 mmHg·min for CVP > 20 mmHg. In the study by Huang et al. [16], it was reported that CVP was significantly higher in patients with severe AKI compared to those without (11.2 mmHg vs. 9.8 mmHg, *p* < 0.001). Using LASSO regression and logistic regression, CVP was identified as a significant risk factor for severe AKI, with an odds ratio of 1.075 (95% CI: 1.051–1.099, *p* < 0.001).

Demirjian et al. [17] found a significant linear relationship between elevated CVP and AKI risk. Patients with low CVP levels were not at increased risk of AKI, even with lower MAP values, whereas elevated CVP was strongly linked to AKI regardless of MAP. It is noteworthy that elevated CVP and a diminished cardiac index exhibited the most pronounced correlation with AKI, thereby underscoring their synergistic effect on the risk of renal impairment. In the research conducted by Gül et al. [18], it was observed that CVP levels were significantly elevated in the high-risk cohort (mean perfusion pressure < 72 mmHg) when juxtaposed with the low-risk cohort (mean perfusion pressure > 72 mmHg). Specifically, the average CVP for the high-risk group (HR-G) was documented as 13.2 ± 2.9 mmHg, in contrast to 10.9 ± 2.5 mmHg for the low-risk group (LR-G). Within the Acute Kidney Injury Network (AKIN) cohort, the mean CVP was recorded at 13.6 ± 3.2 mmHg, as opposed to 11.2 ± 2.6 mmHg in the normal renal function (NRF) group. In the research conducted by Schiefenhövel et al. [19], an optimal mean central venous pressure (miCVP) threshold of 11.3 mmHg, rounded to 11 mmHg, was established for predicting mortality in the Intensive Care Unit (ICU). Individuals presenting with miCVP > 11 mmHg demonstrated a markedly elevated incidence of AKI (69.6%) relative to those with miCVP ≤ 11 mmHg (64.2%). Wang et al. [20] elucidated that elevated CVP was independently associated with cardiac surgery-associated acute kidney injury (CSA-AKI). The mean CVP values were significantly greater in the AKI group when compared to the non-AKI group, with the AKI cohort exhibiting a median CVP of 4 mmHg (interquartile range [IQR]: 1) versus 2 mmHg (IQR: 1) in the non-AKI cohort (*p* = 0.015). These findings indicate that higher CVP levels were associated with increased risk of AKI, highlighting the potential role of CVP as a predictive marker for renal complications in patients.

### 3.2. The Role of Postoperative CVP in Predicting AKI

Postoperative CVP was also extensively examined across several studies to evaluate its role in predicting the development of AKI. McCoy et al. [21] prospectively collected data from 4164 patients who underwent cardiac surgery. They were treated with intravenous loop diuretics and there was at least one measurement of CVP within 24 h of the first ICU admission. Their study proved that the higher the CVP, the higher the development of AKI, with a wide range from 36% in patients with a mean CVP < 9 mmHg to 54% in patients with a mean CVP > 12 mmHg. Moreover, per 1 mmHg increase in mean CVP, the occurrence of AKI was 1.11 times higher, particularly considering the more severe stages of AKI. CVP levels are of major importance when considering patients with stage 2 and stage 3 AKI in relation to mortality, but CVP does not affect mortality in patients with stage 1 AKI. In the study conducted by Jin et al. [22], patients who developed AKI were found to have significantly higher upper and lower limit ranges (ULR and LLR) of postoperative CVP compared to non-AKI patients. Specifically, the ULR of postoperative CVP was 10.7 ± 3.5 mmHg in the AKI group versus 9.8 ± 2.7 mmHg in the non-AKI group (*p* = 0.013). Similarly, the LLR of postoperative CVP was 5.0 ± 2.2 mmHg in the AKI group compared to 4.5 ± 1.8 mmHg in the non-AKI group (*p* = 0.048). In the study by Kotani et al. [23], among the exploratory exposure variables analyzed, time-weighted average CVP was found to be significantly associated with the progression of AKI. Specifically, the modified odds ratio (OR) for the progression of AKI associated with each unit increase in time-weighted average CVP was determined to be 1.12 (95% confidence interval: 1.05–1.20; *p* = 0.0013). These results underscore the significance of persistent elevations in postoperative CVP as a predictive marker of severe AKI. A noteworthy observation from the research conducted by Kang et al. [24] was that they identified postoperative CVP levels below 6 cmH_2_O and hypotensive episodes as the most robust predictors of CSA-AKI. Through logistic regression analysis, the study elucidated 11 pertinent determinants of CSA-AKI, thereby accentuating the vital influence of postoperative hemodynamic metrics, particularly diminished CVP and hypotension, on the risk of AKI.

### 3.3. CVP and the Need for Renal Replacement Therapy

The postoperative requirement for renal replacement therapy (RRT) was also evaluated in relation to CVP levels across a variety of studies. In the investigation undertaken by Jiang et al. [25], a CVP exceeding 10 mmHg at the point of ICU admission was recognized as a significant risk factor for acute kidney injury requiring renal replacement therapy (AKIRRT). The research conducted by Wei et al. [26] encompassed a cohort of 1288 patients. Individuals who experienced CSA-AKI exhibited markedly elevated CVP levels in comparison to those who did not encounter CSA-AKI. Specifically, the CSA-AKI cohort presented with a higher CVP (11.5 vs. 9.7 mmHg, *p* < 0.01) when contrasted with the non-AKI cohort. Each incremental increase in CVP was correlated with heightened odds of both CSA-AKI (OR = 1.56, *p* < 0.01) and postoperative RRT (OR = 1.49, *p* = 0.02). Wang et al. [27] examined 2048 patients categorized into high- (CVP ≥ 6.5 mmHg) and low-CVP (CVP < 6.5 mmHg) groups. The high-CVP cohort demonstrated a substantially greater incidence of AKI (40.24% vs. 35.92%, *p* = 0.045) and RRT (1.75% vs. 0.79%, *p* = 0.049), thereby indicating that elevated CVP is associated with an increased risk of postoperative renal complications. These findings imply that elevated CVP is correlated with an augmented risk of both AKI and the necessity for RRT subsequent to cardiac surgery. Within the cardiac surgery population studied by Vandenberghe et al. [28], a distinct association was noted between increasing CVP and AKI, with AKI manifesting in over 50% of patients when CVP surpassed 14 mmHg. A logistic regression analysis encompassing mean perfusion pressure (MPP) components, MAP, and CVP, indicated that MAP was independently linked with the complete resolution of AKI at stages ≥ 2 (OR = 1.01 per mmHg; 95% confidence interval [CI]: 1.00–1.02; *p* = 0.047). Conversely, CVP did not exhibit a significant association with the complete resolution of AKI at stages ≥ 2 (OR = 1.01; 95% CI: 0.98–1.05; *p* = 0.536). These findings suggest that while higher CVP is linked to AKI occurrence, it may not influence the reversal of AKI once established, in contrast to MPP and MAP, which appear to play more significant roles in recovery.

### 3.4. CVP as a Limited Indicator of AKI

The aforementioned findings are not consistently supported by all studies in the literature. For instance, Barbu et al. [29] conducted an observational study using prospectively registered data. Out of a cohort of 2661 patients who underwent coronary artery bypass grafting and/or valve surgery, 387 patients (14.5%) experienced postoperative AKI. However, they reported no significant differences in CVP between patients who developed AKI and those who did not. Specifically, the CVP during CPB was reported as 4.8 ± 4.6 mmHg in the AKI group and 4.5 ± 4.3 mmHg in the non-AKI group. Li et al. [30] enrolled 230 patients in their study, of whom 53 (23.0%) developed acute kidney injury (AKI), and 11 (4.8%) required Continuous Renal Replacement Therapy (CRRT). Concerning this report, the multivariate regression analysis demonstrated that CVP, as a marker of venous congestion, exhibited strong predictive capability for the increased incidence of CSA-AKI. However, the difference in CVP values between the AKI and non-AKI groups was relatively modest, indicating limited discriminatory power. The median CVP values in the non-AKI and AKI groups were 11.0 mmHg (IQR: 9.0–12.0) and 11.0 mmHg (IQR: 10.0–14.0), respectively. The study of Hu et al. [31] compared CVP levels between patients with and without AKI, finding no significant difference in mean CVP (8.2 ± 4.4 mmHg vs. 7.8 ± 5.7 mmHg, *p* = 0.602). However, in the multivariate analysis, baseline CVP emerged as a significant and independent predictor of AKI after cardiac surgery, with an odds ratio (OR) > 1, highlighting its role in AKI risk assessment. These results underscore the multifactorial nature of AKICS, with baseline CVP playing a contributory role in risk prediction when combined with other clinical variables.

### 3.5. Association Between CVP Levels and AKI Risk—A Forest Plot Analysis

**Figure 3:**
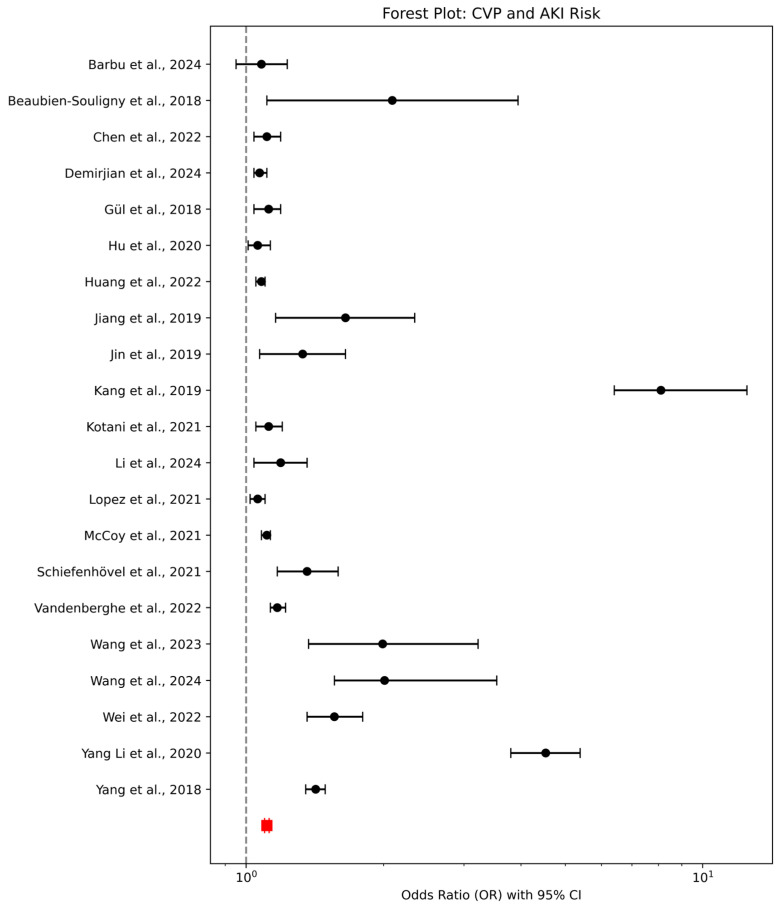
Forest plot analysis of central venous pressure and acute kidney disease. In a forest plot, the diamond is the combined result of all the studies included in this review, showing the overall outcome [11,12,13,14,15,16,17,18,20,21,22,23,24,25,26,27,28,29,30,31].

According to the findings of the 21 studies reviewed, Figure 3 presents a forest plot summarizing the relationship between CVP and the risk of AKI. The plot shows a pooled odds ratio (OR), which suggests a statistically significant association between higher CVP levels and increased risk of AKI, as indicated by a 95% confidence interval (CI) that does not cross 1. Most studies reveal a positive association, reinforcing the idea that elevated CVP contributes to the risk of AKI, particularly in the perioperative and critical care settings. However, there is variability in the strength of this association across studies; for instance, Yang Li et al. [11] reported a strong OR of 4.53, while McCoy et al. [21] reported a more moderate association (OR = 1.11). Additionally, it is important to note that no study demonstrated a significant protective effect from reducing CVP levels in mitigating AKI risk, which could warrant further investigation. The forest plot thus provides a visual summary of these findings, highlighting the overall trend as well as the degree of variability across studies.

## 4. Discussion

### 4.1. Mechanisms Linking Elevated CVP to AKI

Central venous pressure is a crucial parameter in clinical medicine, as it reflects right ventricular preload, which regulates stroke volume through the Frank–Starling mechanism. Elevated CVP can impede venous return, reducing CO and compromising renal perfusion, thereby increasing the risk of AKI [32]. First, high CVP decreases venous return to the right atrium and causes damage to the microcirculatory blood flow, which leads to organ dysfunction [33]. Secondly, a high CVP is transmitted backward, leading to an elevated renal venous pressure, which reduces renal perfusion pressure and increases renal venous congestion [34]. These two factors can cause AKI due to renal dysfunction.

CVP is elevated by either an increase in venous blood volume or by a decrease in venous compliance. Venous return, which is the blood returning to the heart, equals the pressure difference between mean systemic filling pressure and right atrial pressure [35]. Mean Circulatory Filling Pressure (MCFP) is the pressure in the entire circulatory system in the absence of flow. More specifically, Guyton defined MCFP as the hypothetically measured pressure in the circulatory system in the situation that the heart stops instantaneously and the blood is redistributed accordingly, so that there is equality among arterial, capillary, and venous vessel blood pressure. This is usually the pressure people are interested in when they are discussing cardiac preload and vascular function curves, because this is the pressure that is thought to push blood towards the right atrium along a pressure gradient [36]. An elevated CVP or reduced MCFP decreases venous return, which often results in a reduction in CO. Commonly, a decreased CO can lead to a reduction in renal perfusion, which can cause AKI [37]. Additionally, elevated CVP transmits retrogradely to the kidneys, raising renal venous pressure, which reduces renal perfusion pressure, increases venous congestion, and elevates renal vascular resistance. This cascade results in renal hypoperfusion and a significant reduction in glomerular filtration rate (GFR), which is strongly associated with the development of AKI [38].

Several studies confirm these associations. Lopez et al. [15] demonstrated that increased intraoperative venous congestion was independently associated with the development of postoperative AKI. In addition, Demirjian et al. [17] reported that elevated CVP, particularly when combined with a low cardiac index, exhibited the strongest association with AKI. Wei et al. [26] also identified CVP as an independent predictor of renal injury, with higher CVP linked to greater odds of renal dysfunction. Additionally, Li highlighted the predictive ability of CVP for escalating rates of CSA-AKI. Hu et al. [31] further validated the role of baseline CVP as an independent predictor of AKI development, underscoring its relevance in identifying at-risk patients.

Furthermore, an increase in CVP interferes with microcirculatory perfusion by modifying the pressure differential between inflow and outflow pressures, with the latter being contingent upon CVP levels. The probability of AKI and mortality was significantly higher when the CVP was recorded over 8 mmHg due to microcirculatory disruption and decreased RBF [39]. This interference may lead to elevated renal interstitial and subcapsular pressures, thereby intensifying venous congestion and compromising capillary perfusion. Such alterations adversely influence renal autoregulation, an essential physiological mechanism that safeguards against glomerular capillary damage within a restricted MAP range [40]. Importantly, Demirjian et al. [17] identified a notable linear correlation between increased CVP and the risk of AKI, demonstrating that lower CVP levels did not elevate AKI risk even at diminished MAP values, while heightened CVP exhibited a strong association with AKI irrespective of MAP. These observations highlight the critical necessity of sustaining an optimal CVP level to reduce perturbations in renal microcirculation and autoregulation, both of which are indispensable for the proper functioning of the kidneys.

Notably, CVP and renal capillary blood flow are inversely related, and an elevation in CVP leads to reduced capillary blood flow, triggering endothelial stretch and activating pro-inflammatory cytokines. Elevated CVP can also stimulate the renin–angiotensin–aldosterone system (RAAS), induce systemic and renal inflammation, increase endothelial permeability, and elevate sympathetic outflow, compounding the risk of renal dysfunction and AKI. These physiological changes hold significant importance during cardiac surgical procedures, wherein both surgical and CPB-associated influences—such as hemodilution, systemic inflammatory responses, and extended bypass durations—have the potential to intensify the ramifications of increased CVP and augment the likelihood of AKI [41].

### 4.2. Surgical and CPB-Related Factors Influencing CVP and AKI

CPB is recognized for inducing substantial hemodynamic changes, particularly venous congestion, especially in the presence of elevated CVP [27]. This venous congestion intensifies endothelial distension, resulting in endothelial dysfunction and the activation of pro-inflammatory cytokines (e.g., IL-6, TNF-α), which exacerbate the systemic inflammatory response syndrome (SIRS) linked to CPB [42]. The consequent inflammatory response and increased endothelial permeability facilitate systemic capillary leakage, tissue edema, and microcirculatory dysfunction within critical organs such as the kidneys and lungs [42].

Increased CVP during CPB further exacerbates these deleterious effects by diminishing renal perfusion, elevating renal venous pressure, and stimulating the renin–angiotensin–aldosterone system (RAAS). The complex interaction among venous congestion, RAAS activation, and sympathetic nervous system activation establishes a detrimental cycle of inflammation, fluid retention, and elevated oxygen requirements in already compromised tissues [43]. Furthermore, CPB intrinsically provokes systemic inflammation due to exposure to the artificial circuit and ischemia–reperfusion injury, with elevated CVP serving to amplify this inflammatory response. This convergence aggravates renal impairment, organ damage, and adverse clinical outcomes, including AKI [44].

Cardiac stunning is a common phenomenon of temporary cardiac dysfunction, seen frequently in the setting of cardiac surgeries, especially after CABG [45]. Right ventricular function appeared to be more deteriorated than left ventricular function after CABG [46]. The influence of CPB on the functionality of the right ventricle (RV) is of paramount significance, given that the RV plays a crucial role in modulating venous return and facilitating pulmonary circulation. An extended duration of CPB or augmented RV afterload resulting from pulmonary hypertension may precipitate RV dysfunction, thereby diminishing its efficacy in accommodating venous return adequately [47]. Such dysfunction further intensifies venous congestion, leading to an elevation in CVP. As CVP generally indicates right-sided cardiac filling pressure, it may be a helpful indicator of acute RV dysfunction. Although it is a static hemodynamic parameter, the trend of CVP gives important information regarding a patient’s management [48]. Specifically, CVP less than 10 mmHg can almost rule out RV dysfunction with congestion [49]. The combination of CPB-induced systemic inflammation, RV dysfunction, and elevated CVP leads to a significant increase in the risk of AKI.

Efforts to regulate CVP during cardiac surgical procedures are vital, given that elevated CVP exacerbates the inflammatory load imposed by CPB. Approaches such as optimizing fluid management or employing ultrafiltration during CPB may reduce venous congestion, attenuate inflammation, and improve clinical outcomes [9]. Nevertheless, the efficacy of these interventions relies on the establishment of and adherence to optimal CVP thresholds throughout the perioperative phase. Elevated CVP has been identified as a predictive factor of AKI, underscoring the necessity for explicit guidelines on CVP management to mitigate renal injury while preserving hemodynamic stability [9]. This underscores the significance of exploring CVP thresholds as prognostic indicators of AKI in surgical patients.

### 4.3. CVP Thresholds to Predict AKI

The imperative to establish an optimal CVP threshold aimed at minimizing the risk of AKI without adversely affecting other hemodynamic parameters is of paramount importance, as the inconsistency in thresholds observed across various studies and patient demographics underscores the deficiency of a standardized methodology. Legrand et al. [50] documented a linear relationship linking CVP levels to the likelihood of AKI, particularly noted when CVP values are above 2 mmHg. This observation was further validated by Demirjian et al. [17], who similarly recognized a significant linear association between elevated CVP and the risk of AKI. In the present review, a notable heterogeneity in CVP thresholds across different studies is apparent, thereby accentuating the lack of a universally accepted benchmark for the prediction of AKI risk.

It is imperative to ascertain an upper limit for CVP that effectively balances the potential risk of AKI while concurrently ensuring optimal hemodynamic stability. The majority of the literature delineates a CVP threshold of 10 cmH_2_O as an indicator of increased AKI risk. Specifically, Yang et al. [11] elucidated that a CVP exceeding 10 cmH_2_O is correlated with a sixfold augmentation in the risk of AKI, while Li et al. [12] reported an odds ratio of 4.53 for AKI at the identical threshold. In contrast, a more elevated cutoff was utilized in the investigation conducted by Vandenberghe et al. [28], wherein AKI was observed in over 50% of subjects with a CVP surpassing 14 mmHg. Conversely, Wang et al. [27] employed a lower threshold, demonstrating that a CVP measurement of 6.5 cmH_2_O during CPB considerably heightens the postoperative risk of AKI. Intermediate values of CVP have been recognized as pivotal thresholds for the emergence of AKI. In the study by Gül et al. [18], a CVP level exceeding 13.2 mmHg was linked to a substantial risk of AKI, whereas the research conducted by Huang et al. [16] established a more modest threshold of 11.2 mmHg. Likewise, Schiefenhövel et al. [19] indicated that a CVP level of 11 mmHg corresponded to a 69.6% likelihood of developing AKI. The delineation of this upper threshold may serve to inform clinical management strategies and avert adverse clinical outcomes.

Postoperative CVP has been subjected to extensive investigation through a plethora of studies to determine its prognostic significance in relation to the development of AKI. In the research conducted by Jiang et al. [25], a CVP exceeding 10 mmHg upon admission to the ICU was recognized as a critical risk factor for AKI necessitating RRT. Likewise, the research by McCoy et al. [21] indicated that 54% of patients exhibiting a mean CVP greater than 12 mmHg subsequently experienced AKI. Moreover, the inquiry performed by Jin et al. [22] disclosed that the upper threshold of postoperative CVP was recorded at 10.7 ± 3.5 mmHg among patients who manifested AKI. These studies elucidate the paramount role of hemodynamic alterations following surgical procedures, with postoperative CVP emerging as a pivotal variable correlated with AKI.

Considering the existing literature, it remains unclear whether a definitive threshold for a “lowest possible CVP” can be established. Postoperative CVP measurements that fall below 6 cmH_2_O, in conjunction with the presence of hypotension, have been recognized as the most dependable indicators of CSA-AKI [24]. McCoy’s investigation [21] illustrated that a mean CVP of less than 9 mmHg was linked to a 36% likelihood of developing AKI. The aforementioned results accentuate the necessity for judicious management of CVP, as it is imperative to sustain it at a level adequate to guarantee sufficient CO and organ perfusion. In the study conducted by Jin et al. [22], patients who developed AKI also had lower postoperative CVP limit ranges compared to non-AKI patients. Specifically, the lower limit range (LLR) of postoperative CVP was 5 mmHg in the AKI group.

Beyond differences in patient characteristics and surgical techniques, institutional protocols for perioperative hemodynamic monitoring and fluid management may further impact AKI outcomes. For example, some studies included in this review utilized goal-directed therapy strategies, while others relied on standard intraoperative monitoring, which could influence CVP measurements and their predictive value for AKI. Additionally, differences in postoperative care, including the use of diuretics, vasopressors, and renal replacement therapy, may also contribute to variability in AKI incidence and progression. Given these variations, the standardization of CVP thresholds and AKI definitions remains a critical challenge in establishing universally applicable predictive criteria.

In general, for optimal patient outcomes, particularly after cardiac surgery, it is crucial to avoid sustained high CVP levels, as these are associated with an increased risk of complications, including renal injury. Therefore, establishing a clinically appropriate CVP threshold is essential to guide fluid management, minimize hemodynamic instability, and reduce the likelihood of adverse outcomes. Through the analysis of these 21 studies, a clear association between higher CVP and increased AKI risk emerged. The most critical CVP thresholds identified were 10 mmHg, 12 mmHg, 14 mmHg, and 20 mmHg, with risk increasing progressively beyond these values. CVP ≥ 10 mmHg was the most commonly reported cutoff for elevated AKI risk, showing 1.42 to 4.53 times increased odds. CVP ≥ 12 mmHg further amplified the risk, while CVP ≥ 14 mmHg was consistently associated with severe AKI and the need for RRT. The highest threshold (CVP ≥ 20 mmHg) showed the greatest risk escalation, linked to fluid overload, right heart failure, and mortality. Studies also suggest an optimal CVP range of 6–8 mmHg to minimize AKI incidence. Research on the optimal value for CVP is something that future studies should focus on.

### 4.4. Clinical Implications and Strategies for CVP Management in Cardiac Surgery

The changes in CVP (ΔCVP) during different phases of cardiac surgery can be understood as the ratio of the change in blood volume (ΔV) within the thoracic venous system to its compliance (C), expressed as ΔCVP = ΔV/C. An increase in CVP can result from either an increase in blood volume or a decrease in venous compliance. For patients with elevated CVP but no signs of volume overload, maintaining balance is crucial [51]. In these cases, reducing CVP through volume extraction methods such as diuretics or ultrafiltration can be challenging and potentially harmful. McCoy et al. [21] demonstrated that CVP does not predict the risk of AKI associated with diuretic use, as the magnitude of the association between intravenous loop diuretic use (versus non-use) and AKI did not vary across tertiles of CVP. The excessive use of these interventions can lead to adverse effects, including cardiac dysfunction, reduced CO, and impaired RBF. Therefore, the continuous and careful monitoring of CO, CVP, and renal perfusion is essential to avoid both under- and over-treatment. The approach to managing CVP should be tailored to the individual patient, taking into account the underlying condition. Chen et al. [37] suggested that the ideal CVP should be adapted to each patient’s hemodynamic status. For instance, in the context of heart failure and cardiorenal syndrome, enhancing the interactions between the lungs and the right heart may prove to be more advantageous than an aggressive reduction in intravascular volume, thereby underscoring the necessity for a comprehensive and individualized strategy in the management of CVP during cardiac surgical procedures [52].

The management of CVP throughout cardiac surgery is of paramount importance, as an elevated CVP not only exacerbates venous congestion, but also contributes to renal impairment, potentially culminating in AKI and the subsequent requirement for RRT. As previously mentioned, fluctuations in CVP during surgical interventions can be correlated with variations in renal perfusion, and the implications of volume removal or therapeutic measures, such as diuretics and ultrafiltration, on CVP necessitate careful examination to prevent further detriment to renal function. Numerous studies have established that elevated CVP serves as an independent risk factor for AKI and the necessity for RRT following surgical procedures. Specifically, Jiang et al. [25] identified a CVP exceeding 10 mmHg upon admission to the ICU as a significant risk factor for AKI necessitating RRT, while Wei et al. [26] reported that each incremental increase in CVP substantially heightened the likelihood of developing CSA-AKI and postoperative RRT. Furthermore, Wang et al. [28] illustrated that patients exhibiting a CVP of 6.5 mmHg or higher demonstrated a significantly elevated incidence of AKI and were more frequently required to undergo RRT when compared to individuals with lower CVP values.

It is also crucial to address the role of preoperative CVP measurements and their potential impact on the development of postoperative complications, including the occurrence of AKI. Based on the studies that were included in this review, higher preoperative CVP is associated with increased risk of AKI (OR: 1.12, 95% CI: 1.06–1.11). Preoperative CVP appears to be a slightly stronger predictor of AKI than postoperative CVP, but the difference is not statistically significant (*p* = 0.069). High CVP before surgery may indicate poor venous return or impaired right heart function, which can lead to insufficient renal perfusion. If the kidneys are not adequately perfused, there is an increased risk of ischemic damage to the renal tissues, which can contribute to the development of AKI. Additionally, elevated preoperative CVP may reflect an underlying volume overload or a condition like heart failure, which itself is a risk factor for AKI. High CVP could impair the ability to maintain an optimal balance between fluid status and renal perfusion during surgery, thereby increasing the likelihood of AKI postoperatively [53]. Furthermore, preoperative high CVP may suggest altered hemodynamic parameters or cardiovascular dysfunction that may persist during and after surgery, contributing to poor organ perfusion and potentially exacerbating renal injury [54]. The relationship between preoperative CVP and AKI remains an area of ongoing research, and further studies are needed to define specific thresholds and interventions for high CVP as a preoperative risk factor.

### 4.5. The Impact of Comorbidities on AKI Risk and CVP Levels

Individuals with previously diagnosed chronic kidney disease, diabetes mellitus, and heart failure have been discerned as possessing the greatest probability for the onset of AKI. These medical conditions are recognized as significant risk determinants that independently elevate the probability of renal complications subsequent to cardiac surgical procedures or other medical interventions [55]. In the studies reviewed, patients with chronic renal disease or those on dialysis were excluded. This exclusion was implemented to minimize potential confounding factors and to ensure a more focused evaluation of the relationship between CVP levels and the development of AKI in patients undergoing cardiac surgery. While this may limit the generalizability of the findings to populations with pre-existing renal conditions, it enhances the study’s specificity in assessing the role of CVP in AKI risk among a more homogenous patient group.

Comorbidities play a significant role in influencing CVP levels, as various underlying conditions can affect venous return, circulatory volume, and cardiac function. For instance, patients with heart failure, fluid overload, or conditions leading to increased venous pressure may experience elevated CVP. Specifically, those with pulmonary hypertension or right heart failure often have compromised right ventricular function, leading to increased systemic venous pressures and higher preoperative CVP values [56]. These elevated CVP levels can impair renal perfusion by affecting renal venous return and glomerular filtration, thereby increasing the risk of developing AKI. The presence of such comorbidities should be carefully considered when evaluating the association between CVP and AKI risk, as these conditions may independently contribute to renal dysfunction, potentially complicating the interpretation of CVP as a predictor of AKI.

### 4.6. Limitations

This study has several limitations that must be acknowledged. The included studies exhibit heterogeneity in study design, CVP thresholds, and AKI definitions, which may influence the consistency of the findings. The included studies varied in design, with some being prospective and others retrospective; however, all were observational in nature. The heterogeneity observed in the CVP thresholds across various investigations may be ascribed to multiple determinants. Variations in patient cohorts, encompassing pre-existing comorbidities and demographic variables, could significantly affect the association between CVP and the risk of AKI [57]. Furthermore, discrepancies in research design, including the methods employed for measuring CVP, the temporal aspects of these measurements, and the approaches utilized for evaluating AKI, could have resulted in divergent thresholds. The existence of confounding variables, such as fluid management strategies, hemodynamic stability, and additional organ dysfunctions, may also have played a role in the absence of a universally recognized CVP threshold for the prediction of AKI [58]. An analysis of the 21 reviewed studies revealed that AKI was defined using various classification systems. AKIN classification was used by Gül et al. [18] and RIFLE classification was used by Hu et al. [31], while in the rest of the studies, KDIGO classification was used. Variability in AKI classification may explain the variation in CVP cutoffs that are reported. Variability in AKI thresholds may affect the interpretation of CVP cutoffs due to different sensitivity levels. RIFLE and AKIN use slightly different serum creatinine and urine output cutoffs, leading to variations in how early AKI is detected. KDIGO is more sensitive, incorporating smaller creatinine increases, meaning some studies may have reported a higher AKI incidence at lower CVP thresholds [59]. Studies using KDIGO criteria tended to report lower CVP thresholds (≥10 mmHg), while RIFLE/AKIN-based studies reported slightly higher CVP cutoffs (≥12–14 mmHg). Future studies should standardize AKI definitions to improve comparability. Additionally, the lack of long-term outcome data, such as CKD progression and mortality, limits the ability to assess the broader implications of AKI following cardiac surgery. While these studies establish an association between elevated CVP and AKI in the perioperative period, they do not provide insight into whether AKI episodes lead to persistent renal dysfunction or increased long-term mortality. Given that AKI is a known risk factor for CKD and adverse cardiovascular outcomes, future research should focus on longitudinal studies assessing renal recovery, CKD incidence, and overall survival following AKI in patients undergoing cardiac surgery. Another limitation of this review is the presence of publication bias, as suggested by the funnel plot asymmetry and Egger’s test results. This bias may arise from the selective publication of studies reporting significant associations between CVP and AKI, potentially overestimating the true effect size. Future research should aim to address this limitation by including unpublished data and conducting further sensitivity analyses.

### 4.7. Gaps in the Research and Future Directions

While the existing literature regarding CVP and its prognostic value in forecasting AKI predominantly focuses on critically ill patients, there exists a notable deficiency in the transference of this knowledge to the field of cardiac surgery, an area characterized by distinct challenges. The repercussions of CPB, which include inflammatory responses, fluid redistribution, and venous congestion, may intensify the adverse effects of elevated CVP on renal functionality. Subsequent investigations ought to attempt to more effectively combine CVP with dynamic parameters or biomarkers to enhance the prognostication of AKI, especially in surgical scenarios where such factors are of paramount importance. This may encompass the implementation of advanced monitoring methodologies, such as continuous CO records, to evaluate real-time hemodynamic fluctuations. Moreover, the integration of biomarkers, including urinary indicators (e.g., neutrophil gelatinase-associated lipocalin [NGAL] and kidney injury molecule-1 [KIM-1]) or plasma markers (e.g., cystatin C), in conjunction with CVP assessments could potentially augment early identification and improve the risk stratification of AKI [60]. Longitudinal investigations that examine the temporal variations in CVP and its correlation with these dynamic parameters could yield a deeper understanding regarding the manner in which CVP fluctuations contribute to renal impairment.

While CVP is recognized as a factor associated with AKI, it is not commonly included as a standalone variable in existing AKI prediction scoring systems. High CVP levels have consistently been associated with AKI risk, and integrating CVP into a predictive algorithm could be valuable. However, CVP alone may not be sufficient due to its dynamic nature and dependence on multiple factors, such as volume status, cardiac function, and vascular tone. Some newer models incorporate hemodynamic parameters, but the role of CVP as an independent predictor in these algorithms remains an area for further research. Future studies could explore how CVP, in combination with other dynamic parameters (e.g., mean perfusion pressure, renal biomarkers, and fluid responsiveness), might enhance the accuracy of AKI prediction models in perioperative settings.

Furthermore, the incorporation of machine learning algorithms or predictive modelling techniques may facilitate the establishment of individualized thresholds, thereby enhancing the applicability of CVP in forecasting AKI, particularly within the intricate perioperative and postoperative areas of cardiac surgery. Through the synthesis of CVP with these advancing technologies, the capability to anticipate and avert AKI in patients undergoing cardiac surgery may be markedly enhanced, ultimately leading to improved patient outcomes.

## 5. Conclusions

Elevated CVP has been recognized as an accurate indicator for the onset of AKI in patients undergoing cardiac surgical procedures. These observations highlight the potential importance of CVP monitoring in forecasting AKI and guiding perioperative management protocols. Considering the pivotal role of CVP in renal perfusion, it is imperative to implement a tailored approach to enhance its management, particularly focusing on sustaining CVP within a constrained range to alleviate the risk of renal impairment. The continuous observation of CVP is crucial for the assessment of the probability of AKI in order to achieve early prevention and individualized treatment for renal complications.

## Figures and Tables

**Table 1:** KDIGO stages of AKI.

Stage	Serum Creatinine	Urine
1	1.5–1.9 times baseline OR ≥0.3 mg/dL (≥26.5 μmol/L) rise	<0.5 mL/kg/h for 6–12 h
2	2.0–2.9 times baseline	<0.5 mL/kg/h for ≥12 h
3	3.0 times baseline OR Increase in serum creatinine ≥ 4.0 mg/dL OR inflation of RRT OR Decrease in eGFR to <35 mL/min per 1.73 m^2^ (patients < 18 years)	<0.3 mL/kg/h for ≥24 h OR Anuria for ≥12 h

Abbreviations: KDIGO: Kidney Disease Improving Global Outcomes, AKI: acute kidney injury, RRT: renal replacement therapy, eGFR: estimated glomerular filtration rate.

**Table 2:** RIFLE stages of AKI.

Stage	Serum Creatinine	GFR Criteria	Urine Output
Risk (R)	1.5 times baseline	>25% decrease	<0.5 mL/kg/h for 6 h
Injury (I)	2.0 times baseline	>50% decrease	<0.5 mL/kg/h for 12 h
Failure (F)	3.0 times baseline OR creatinine ≥ 4 mg/dL with acute rise ≥ 0.5 mg/dL	>75% decrease	<0.3 mL/kg/h for 24 h OR anuria for 12 h
Loss (L)	Persistent AKI with complete loss of kidney function for >4 weeks		
ERSD (E)	Complete loss of kidney function for >3 months		

Abbreviations: AKI: acute kidney injury, GFR: glomerular filtration rate.

**Table 3:** AKIN stages of AKI.

Stage	Serum Creatinine	Urine
1	≥0.3 mg/dL (≥26.4 μmol/L) rise or 150–200% increase from baseline	<0.5 mL/kg/h in 6 h
2	1.5 times baseline OR ≥50% increase	<0.5 mL/kg/h in 12 h
3	3.0 times baseline OR Increase in serum creatinine ≥ 354 μmol/L, with an acute increase by at least 44 μmol/L OR inflation of RRT OR	<0.3 mL/kg/h in 24 h OR Anuria for 12 h

Abbreviations: AKIN: Acute Kidney Injury Network, AKI: acute kidney injury, RRT: renal replacement therapy.

**Table 4:** Cleveland Clinic Score for predicting cardiac surgery-associated AKI.

Risk Factor	Points Assigned
Female sex	1
COPD requiring treatment	1
Congestive heart failure	2
LVEF < 35%	2
Diabetes mellitus and insulin treatment	1
Preoperative creatinine > 1.2 mg/dL	2
Use of intra-aortic balloon pump	2
CPB time > 120 min	3
Reoperation	2

The Cleveland Clinic Score categorizes patients into three risk groups based on their total score. Patients with a score between 0 and 2 points are classified as low risk, with a relatively low probability of developing AKI. Those with a score of 3 to 5 points fall into the intermediate-risk category, indicating a moderate likelihood of AKI occurrence. Finally, patients scoring 6 or more points are considered high risk, with a significantly increased probability of postoperative AKI. Abbreviations: LVEF: left ventricular ejection fraction, COPD: Chronic Obstructive Pulmonary Disease, CPB: cardiopulmonary bypass.

**Table 5:** Mehta Score for predicting cardiac surgery-associated AKI.

Risk Factor	Points Assigned
Age ≥ 65 years	2
Emergency surgery	1
Congestive heart failure	2
LVEF < 35%	2
Preoperative creatinine > 1.2 mg/dL	2
Use of intra-aortic balloon pump	2
CPB time > 120 min	3
Reoperation	2

Based on the total score, patients are categorized into risk levels. A score of 0–5 indicates low risk, 6–9 represents moderate risk, and a score of 10 or higher signals high risk for AKI. Abbreviations: LVEF: left ventricular ejection fraction, CPB: cardiopulmonary bypass.

**Table 6:** Literature data concerning the association between CVP and AKI.

Study, Year	Country	Design	Population Size	AKI Classification	NOS Score	CVP Threshold
Yang et al., 2018, [11]	China	Prospective Study	1941 patients underwent cardiac surgery	KDIGO	7 stars	CVP > 10 cmH_2_O at the end of surgery
Li et al., 2020, [12]	China	Prospective Study	5533 participants underwent cardiac surgery	KDIGO	8 stars	Postoperative CVP of ≥10 mmHg
Beaubien-Souligny et al., 2018, [13]	Canada	Prospective Observational Cohort Study	145 patients undergoing cardiac surgery	KDIGO	6 stars	Higher CVP measurements at the end of surgery were associated with AKI
Chen et al., 2022, [14]	China	Retrospective Cohort Study	5127 patients undergoing cardiac surgery	KDIGO	6 stars	Intraoperative venous congestion above 16 or 20 mmHg resulted in a 7% or 11% increase in AKI risk
Lopez et. al., 2020, [15]	USA	Prospective Study	435 patients undergoing cardiac surgery	KDIGO	6 stars	Increased intraoperative venous congestion was independently associated with the development of postoperative AKI
Huang et al., 2022 [16]	China	Retrospective Cohort Study	6271 patients admitted to the CSRU	KDIGO	9 stars	CVP of 11.2 mmHg in the severe AKI group (*p* < 0.001)
Demirjian et al., 2024, [17]	Cleveland, OH	Retrospective Observational Study	40,426 patients undergoing cardiac surgery	KDIGO	7 stars	Elevated CVP and low cardiac index had the strongest association with AKI
Gül et al., 2018, [18]	Cyprus	Prospective Study	147 patients undergone TAVI procedure	AKIN	8 stars	The mean value of CVP was 13.2 ± 2.9 mmHg in HR-G vs. LR-G (10.9 ± 2.5 mmHg)
Schiefenhövel et al., 2021, [19]	Germany	Retrospective Observational Cohort Study	9802 patients	KDIGO	7 stars	High CVP group (>11 mmHg) had a higher incidence of AKI
Wang et al., 2023, [20]	China	Retrospective Study	15,387 patients undergoing cardiac surgery	KDIGO	9 stars	The average CVP values were significantly higher in the AKI group (CVP of 4 mmHg) compared to the non-AKI group (CVP of 2 mmHg)
McCoy et al., 2020, [21]	California	Retrospective Cohort Study	4164 post-cardiac surgical patients	KDIGO	6 stars	CVP > 12 mmHg associated with 54% probability of AKI
Jin et al., 2019, [22]	China	Retrospective Study	300 patients underwent cardiac surgery	KDIGO	7 stars	The peak value of postoperative CVP independently contributed to the development of AKI
Kotani et al., 2021, [23]	Japan	Retrospective Study	746 patients undergoing cardiac procedure	KDIGO	8 stars	CVP of 9.4 cmH_2_O at the AKI group
Kang et al., 2019, [24]	China	Retrospective Cohort Study	1468 patients undergoing cardiac surgery	KDIGO	7 stars	CVP < 6 cmH_2_O was important clinical factor that increased the risk of post-operative AKI
Jiang et al., 2019, [25]	China	Retrospective Case–control Study	1773 cardiac surgery patients	KDIGO	7 stars	CVP > 10 mmHg is a predictor of AKI-RRT
Wei et al., 2022. [26]	United States	Retrospective Cohort Study	1288 patients undergoing cardiac surgery	KDIGO	7 stars	Patients who developed CSAKI had mean CVP of 11.5 mmHg
Wang et al., 2024, [27]	China	Retrospective Study	2048 patients undergoing cardiac procedure	KDIGO	8 stars	Elevated CVP (≥6.5 cmH_2_O) in CPB during cardiac operation is associated with an increased risk of AKI
Vandenberghe et al., 2022, [28]	Belgium	Retrospective Cohort Study	3415 patients undergoing cardiac surgery	KDIGO	8 stars	CVP above 14 mmHg associated with AKI
Barbu et al., 2023, [29]	Sweden	Observational Study	2661 coronary artery bypass grafting and/or valve patients operated on	KDIGO	7 stars	There were no significant differences between AKI and non-AKI patients in terms of CVP
Li et al., 2024, [30]	China	Prospective Observational Cohort Study	230 patients undergoing cardiac surgery	KDIGO	7 stars	CVP exhibited strong predictive ability for the escalation of CSA-AKI rates
Hu et al., 2020. [31]	Australia	Retrospective Observational Study	513 patients undergoing CPB	RIFLE	5 stars	Confirmation of the association of baseline CVP as an independent predictor of the development of AKICS

Abbreviations: CVP: central venous pressure, AKI: acute kidney injury, NOS: Newcastle–Ottawa scale, CSA-AKI: cardiac surgery-associated acute kidney injury, AKICS: Acute Kidney Injury after Cardiac Surgery, RRT: renal replacement therapy, HR-G: high-risk group, LR-G: low-risk group, AKIN: Acute Kidney Injury Network.

## Data Availability

The original contributions presented in this study are included in the article.
